# A Case Report in Hemorrhagic Stroke: A Complex Disease Process and Requirement for a Multimodal Treatment Approach

**DOI:** 10.7759/cureus.2976

**Published:** 2018-07-13

**Authors:** Brain D Sindelar, Vimal Patel, Shakeel Chowdhry, Julian E Bailes

**Affiliations:** 1 Neurosurgery, University of Florida, Gainesville, USA; 2 NorthShore Neurological Institute, NorthShore University Health System/University of Chicago Pritzker School of Medicine, Chicago, USA; 3 Neurosurgery, NorthShore University Health System/University of Chicago Pritzker School of Medicine, Chicago, USA

**Keywords:** intracranial hemorrhages, cerebral hemorrhage, hematoma, thrombolytic therapy, stroke

## Abstract

Intracerebral hemorrhage (ICH) with or without intraventricular hemorrhage (IVH) is a highly morbid disease process due to the mass effect and secondary injury that occurs upon the surrounding brain. Historically, surgical evacuation has failed to demonstrate improved outcomes in comparison to standard medical therapy likely due to the significant brain trauma when accessing the clot. Recent minimally invasive techniques have proposed a way to improve outcomes by reducing this injury. We report here a 62-year-old male with ICH and IVH with acute neurological deterioration due to hydrocephalus was found to have no improvement following external ventricular drainage. A repeat non-contrasted computed tomography (CT) head was obtained which demonstrated the worsening mass effect from peri-hematoma edema. Surgical intervention was employed that uses a variety of techniques (endoscopic and exoscopic visualization, stereotactic trans-sulcal approach and side cutting aspiration, and intraventricular thrombolytic therapy) to reduce cerebral trauma while effectively removing both ICH and IVH. The surgical intervention reduces the mass effect and associated secondary injury, lessens the likelihood of shunt placement and length of stay, and improves long-term morbidity. We conclude that the effectiveness of surgical management of ICH could potentially be improved by employing a multifaceted approach to address the different characteristics of the hemorrhagic stroke.

## Introduction

Minimally invasive surgical interventions for evacuation of intracerebral hemorrhage (ICH) and/or intraventricular hemorrhage (IVH) (in order to remove mass effect, prevent secondary injury, and potentially reduce morbidity/mortality) have demonstrated a range of published clinical outcomes, and therefore the use of one specific or any surgical modality is greatly contested. Here, we will present our management of a particular case of significant ICH with IVH with the purpose of transitioning the dialogue away from choosing a single medical or specific surgical approach to suggesting a multifaceted treatment tactic of ICH in order to reduce this devastating affliction.

## Case presentation

**Figure 1:**
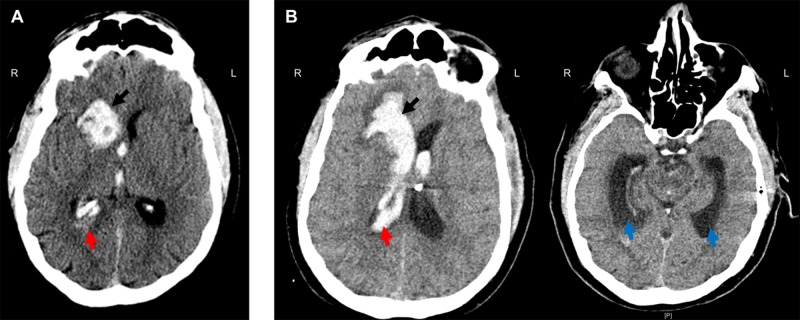
Head computed tomography (CT) pre- and post-admission. A) Right caudate intracerebral hemorrhage (ICH) (17 cm^3^) (black arrow) with ventricular extension (red arrow) but without hydrocephalus. B) Repeat CT head significant for slight increased size of ICH (21 cm^3^) (black arrow) with greater intraventricular hemorrhage (red arrow), casting of the right lateral and third ventricles, and hydrocephalus (blue arrow).

A 62-year-old male with a history of gastroesophageal reflux and deep vein thrombosis/pulmonary embolism, developed sudden onset headache prior to his scheduled Nissen fundoplication. The patient presented to an outside hospital neurologically intact, but due to intractable symptoms, a non-contrasted head computed tomography (CT) was ordered which was significant for a right-sided caudate ICH with ventricular extension but without hydrocephalus (Figure 1A) (ICH score 1). Of note, the patient’s coagulation labs were within normal range.

**Figure 2:**
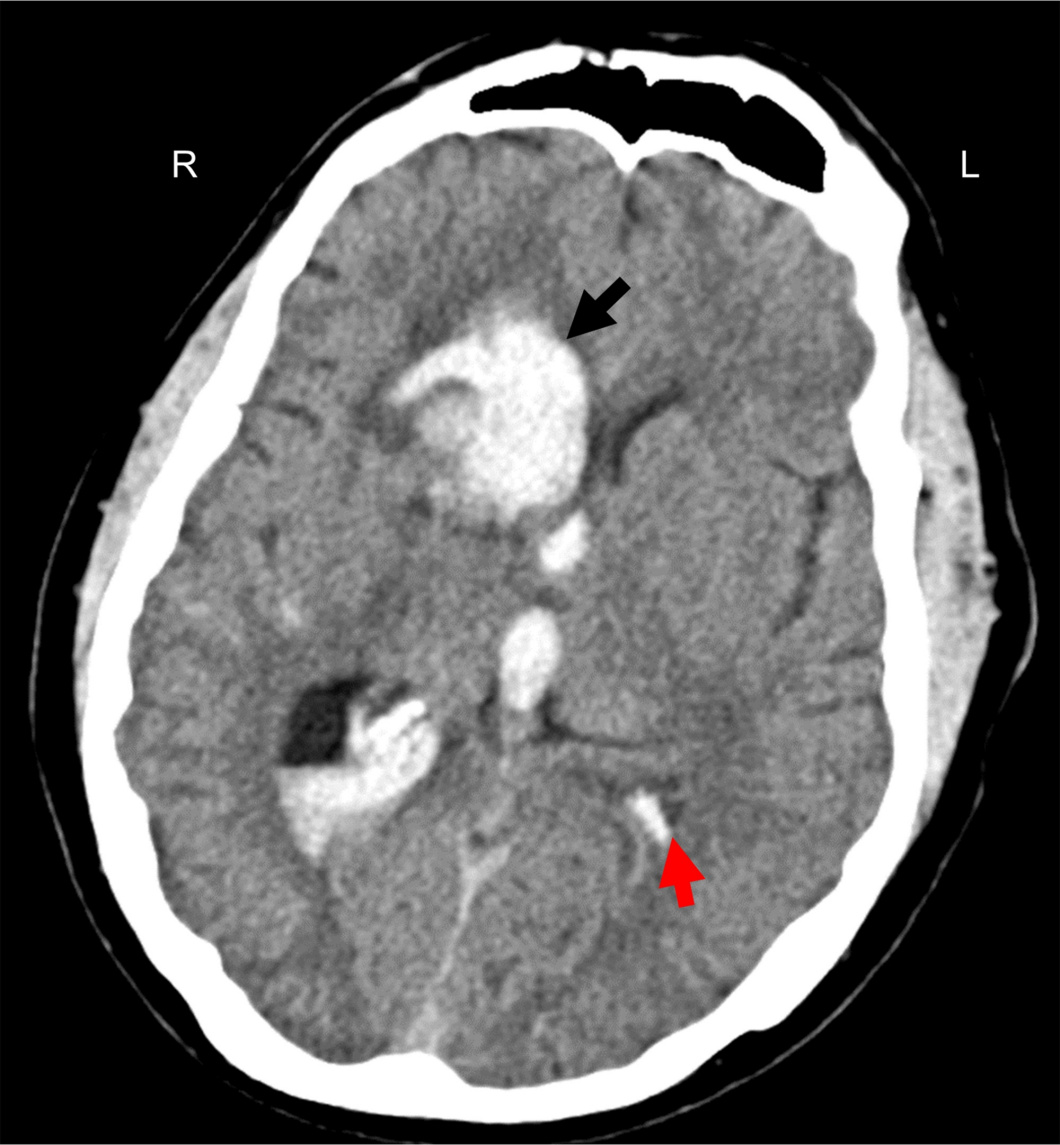
Repeat computed tomography (CT) head six hours (following morning) after presentation. Increased size of the intracerebral hemorrhage (23 cm^3^), a collapsed left lateral ventricle (red arrow) secondary to cerebrospinal fluid drainage, entrapment of the left temporal horn, and peri-hematoma edema with 6.5 mm of midline shift.

En route to our hospital, the patient declined dramatically requiring intubation upon arrival. Repeat imaging was significant for expansion of the ICH with worsening of the IVH and associated hydrocephalus (Figure 1B). The patient was localizing on the right upper extremity and withdrawing in the left upper extremity and bilateral lower extremities to noxious stimuli (GCS 7t, ICH score 2). An external ventricular drain (EVD) was placed and the patient was admitted to the intensive care unit (ICU). Vascular imaging was negative for underlying malformations. A repeat CT head six hours post EVD placement demonstrated a collapsed ventricle secondary to cerebrospinal fluid (CSF) drainage, but the progression of perihematoma edema and midline shift (Figure 2). With increasing mass effect and failure of neurological improvement with CSF drainage, it was decided to take the patient to the operating room for ICH evacuation.

**Figure 3:**
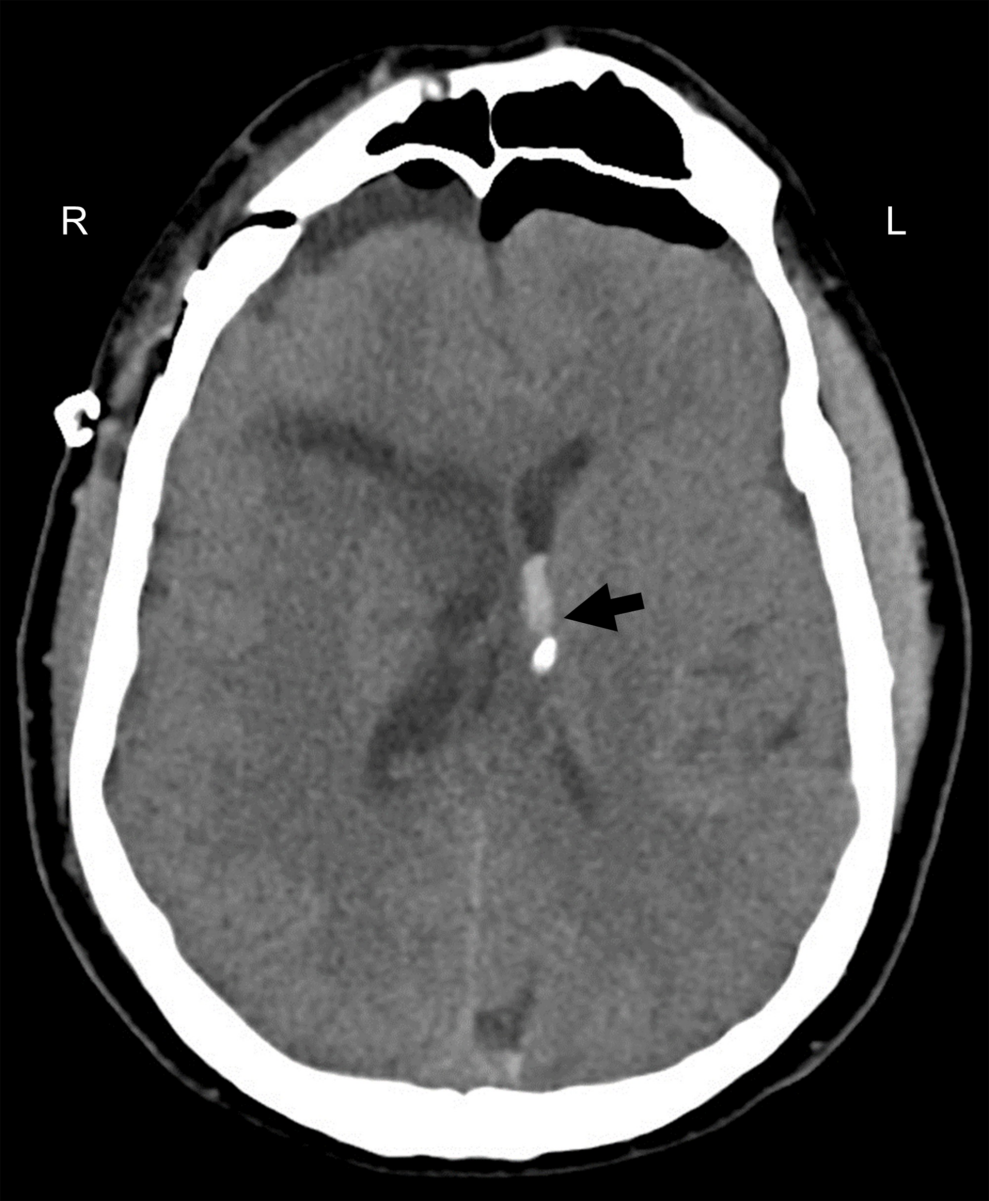
Post-operative head computed tomography (CT). Almost complete removal of the intracerebral hemorrhage and intraventricular hemorrhage from the right lateral ventricle but with persistent clot within the left lateral and third ventricles.

Following anesthetization, a 5 cm curvilinear right frontal incision was made behind the hairline. A 4 cm craniotomy was performed followed by identification of the posterior aspect of the right frontal superior sulcus, and then stereotactic trans-sulcal introduction of a 75 mm sheath and obturator (BrainPath, NICO Corp, Indianapolis, Indiana). Under exoscope magnification, the inferior depth of the hematoma was evacuated with gentle irrigation and suction. A small opening into the right lateral ventricle was identified, and a straight rigid endoscope was used to atraumatically enter the ventricle for further ventricular clot evacuation and irrigation. The endoscope was removed and the trans-sulcal port was slightly retracted in successive fashion to deliver more of the frontal ICH into view. With the use of suction, irrigation, and a side cutting resection device (Myriad, NICO Corp, Indianapolis, Indiana), the remainder of parenchymal hematoma was extracted. A post-operative head CT showed near complete removal of the ICH and IVH from the right lateral ventricle but with residual hematoma predominantly within the left lateral and third ventricles (Figure 3).

Due to the persistence of third ventricle IVH, the patient received intrathecal tPA post-operative day two (two doses, 1 mg each, nine hours apart) followed by successful weaning and removal of the ventricular drain. After three days, the patient was discharged to a long-term acute care hospital. At the three-month follow-up visit, the patient had transitioned to a skilled nursing facility. At the five-month follow-up visit, the patient was living at home, neurologically intact except for a slight facial droop and mild gait imbalance with a goal to return to work in the coming month. At each follow-up, head CT at each outpatient visit was negative for hydrocephalus.

## Discussion

It has been long hypothesized and studied in pre-clinical models that surgical evacuation of an intracerebral hemorrhage aids in removing both the mass effect of the primary injury along with the reduction in the secondary injury associated with clot-induced blood-brain barrier breakdown, the release of inflammatory cytokines, and the development of perihematomal edema. But, two randomized clinical trials, STITCH and STITCH 2, failed to prove this theory due to their inability to strongly demonstrate a statistically significant difference in those offered surgical evacuation through a standard craniotomy compared with medical management, even in those with superficially located lesions [1]. Lack of clinical efficacy has been suggested to be due to the overall morbidity associated with large craniotomies and the cerebral trauma required to access deep-seeded lesions.

For this reason, minimally invasive techniques have been suggested in order to reduce the morbidity associated with a craniotomy, specifically the cerebral injury when retrieving the hematoma. These approaches can be subdivided into those that use thrombolytic agents or those that use mechanical methods for ICH/IVH evacuation. The most popularized approach to targeting intracerebral hemorrhage through thrombolytic means is by stereotactically aspirating the hematoma followed by infusion of either alteplase or urokinase. A phase two clinical trial for this method called “Minimally invasive surgery plus alteplase in intracerebral hemorrhage evacuation”, MISTIE, demonstrated efficacy in reducing clot burden and perihematomal edema and a correlation between outcomes and volume of clot removed [2]. The phase three trial has completed subject enrollment but study results are pending collection of final patient follow-up assessments. A thrombolytic therapy to address IVH has been studied in the “Clot lysis evaluating accelerated resolution,” or CLEAR. This study administered 1 mg of tPA through a ventricular drain every eight hours. This technique reduced overall clot burden but showed no difference in overall outcomes in comparison to control (saline ventricular injection) [3].

Variations in mechanical means to remove deep-seeded clots vary by the type of optics used (endoscope vs exoscope), ways to gain access to the clot, and also various devices to mechanically remove the clot. Case series and retrospective reviews have demonstrated efficacy in clot removal through endoscopic means, but there is only limited evidence demonstrating superior means to standard therapy (medical management). A newer technology published, “minimally invasive subcortical parafascicular access for clot evacuation” or MISPACE, uses an imaged guided placement of a trans-sulcal port with the use of a side cutting aspirator [4]. This approach allows a small craniotomy, practically atraumatic trans-sulcal access to the clot, minimal retraction to surrounding brain, and elimination of pulling that occurs with standard suction. There is currently a randomized multicenter trial (ENRICH: Early Minimally-Invasive Removal of ICH) evaluating the effectiveness of this method but results are still pending [5].

These techniques all bring great promise to possibly improving the standard of care, but due to limited trials directly comparing each to the other and medical management, there is great controversy to their role in ICH management. Due to the multidimensional presentations (size/location of ICH, presence or absence of IVH, etc.) of those with hemorrhagic stroke, we presented this case study in order to pose that a single surgical approach may not be the answer to improving outcomes in comparison to medical management but actually a transition to a multimodal manner that combines the various mechanical and thrombolytic methods to improve visualization, intracerebral and intraventricular clot retrieval, and weaning from ventricular drainage. It appears disadvantageous to not use our arsenal of techniques to provide individualized care to the heterogeneous nature of hemorrhagic stroke. Also, as this case report emphasizes, the standard heterogeneous clinical presentation and management is far different than the homogenous subject pool with two-arm treatment approach standard in clinical trials potentially influencing and explaining the limited benefit demonstrated in previous trials.

## Conclusions

The presentation of a hemorrhagic stroke is multidimensional due to the size, location, and extent of secondary pathological processes (peri-hematomal edema, ventricular hemorrhage, hydrocephalus, etc.). This case report demonstrates the typical clinical presentation of an ICH and demonstrates a multimodal approach that involves not only the standard medical therapy of intracerebral hemorrhage, but also a combination of the various minimally invasive surgical, mechanical and thrombolytic methods in order to safely access and visualize both intracerebral and intraventricular clot to improve removal with the ultimate goal to potentially improve long-term functional outcomes and reduce the requirement of shunting. Though we understand the inherent limitations to an anecdotal case report, we hope that this brings contemplation and consideration in future clinical trials.
